# Serum MicroRNAs Are Promising Novel Biomarkers

**DOI:** 10.1371/journal.pone.0003148

**Published:** 2008-09-05

**Authors:** Shlomit Gilad, Eti Meiri, Yariv Yogev, Sima Benjamin, Danit Lebanony, Noga Yerushalmi, Hila Benjamin, Michal Kushnir, Hila Cholakh, Nir Melamed, Zvi Bentwich, Moshe Hod, Yaron Goren, Ayelet Chajut

**Affiliations:** 1 Rosetta Genomics Ltd., Rehovot, Israel; 2 Division of Maternal Fetal Medicine, Rabin Medical Center, Sackler Faculty of Medicine, Tel Aviv University, Petah-Tiqva, Israel; Texas Tech University Health Sciences Center, United States of America

## Abstract

**Background:**

Circulating nucleic acids (CNAs) offer unique opportunities for early diagnosis of clinical conditions. Here we show that microRNAs, a family of small non-coding regulatory RNAs involved in human development and pathology, are present in bodily fluids and represent new effective biomarkers.

**Methods and Results:**

After developing protocols for extracting and quantifying microRNAs in serum and other body fluids, the serum microRNA profiles of several healthy individuals were determined and found to be similar, validating the robustness of our methods. To address the possibility that the abundance of specific microRNAs might change during physiological or pathological conditions, serum microRNA levels in pregnant and non pregnant women were compared. In sera from pregnant women, microRNAs associated with human placenta were significantly elevated and their levels correlated with pregnancy stage.

**Conclusions and Significance:**

Considering the central role of microRNAs in development and disease, our results highlight the medically relevant potential of determining microRNA levels in serum and other body fluids. Thus, microRNAs are a new class of CNAs that promise to serve as useful clinical biomarkers.

## Introduction

Specific clinical biomarkers have the potential to revolutionize diagnosis and treatment of various medical conditions, ranging from abnormal pregnancies to myocardial infarctions and cancer. In particular, a theme of current cancer research is the quest for sensitive biomarkers that can be exploited to detect early neoplastic changes. Ideally, biomarkers should be easily accessible such that they can be sampled non-invasively. Therefore biomarkers that can be sampled from body fluids, such as serum or urine, are particularly desirable. In recent years it has become clear that both cell-free DNA and mRNA are present in serum, as well as in other body fluids, and that these CNAs represent potential biomarkers [1]–[3]. Accordingly, we considered it likely that microRNAs might be present in bodily fluids.

MicroRNAs are a recently discovered class of small non-coding RNAs that regulate gene expression and have a critical role in many biological and pathological processes [4], [5]. In general, microRNAs are regulated and transcribed like protein coding genes. Subsequent microRNA biogenesis involves discrete processing and transport steps, whereby the active moiety of 20–22 nucleotides is excised from a longer RNA precursor that exhibits specific hairpin structure. Finally, these 20–22 nucleotides are incorporated into a composite machinery, which promotes partial duplex formation between the short RNA and the 3′ untranslated regions (UTR) of targeted mRNAs, resulting typically in mammals in translational silencing [6], [7]. A relevant, though at present enigmatic, feature of microRNA biology is their remarkable stability. For example, microRNAs are preserved well in tissue samples even after formalin-fixation and paraffin-embedding and can be efficiently extracted and evaluated [8]. Therefore, if microRNAs are indeed circulating, we hypothesized further that they should preserve their stability in body fluids. Monitoring the typically small amounts of CNAs in body fluids requires complex and sensitive extraction and detection methods, which until now have been prohibitive either practically or economically [9]. However, we anticipated that the stability of microRNAs should allow development of practicable detection methods, such that they can realistically serve as clinical biomarkers.

Although the total number of microRNAs remains controversial [4], [10], [11] and the roles of specific microRNAs are only beginning to be defined, microRNA expression analyses indicate that diverse tumors display microRNA expression profiles (for mature and/or precursor microRNAs) significantly different from normal tissue [12], [13]. Furthermore, microRNAs are emerging as highly tissue-specific biomarkers [14], [15] with potential clinical applicability for defining the cancer origin of metastases, as we have shown recently [16]. These data led us to expect that the microRNA abundance profile of bodily fluids might reflect physiological and/or pathological conditions and, furthermore, might do so more accurately than an mRNA abundance profile. For mRNA must be translated into protein to have a biological effect whereas microRNAs are themselves the active moiety, often influencing the expression of multiple other genes, and thus likely reflect altered physiology more directly.

Here we validate that microRNAs are present in body fluids and moreover, demonstrate that microRNA levels in serum reflect altered physiological conditions, such as pregnancy.

## Materials and Methods

### Serum samples

Serum samples were collected from 30 women: 10 in the first pregnancy trimester (6–12 weeks of gestational age), 10 in the third pregnancy trimester (34–41 weeks of gestational age) and 10 age-matched non-pregnant women. Eligibility for the study was limited to normal uncomplicated singleton pregnancies with no known fetal malformation. All women provided written informed consent and the local institutional review board approved the study. 8 ml of blood was collected from each woman directly into serum collection tubes (Greiner Bio-one, VACUETTE® Serum Tubes 455071). The whole blood was allowed to stand for about 1 h at RT before being centrifuged at 1800 g for 10 minutes at RT. The resultant serum was aliquoted into eppendorf tubes and stored at −80°C.

### Urine Samples

About 4 ml of urine samples were collected from each individual in a urine container. The urine was then aliquoted into eppendorf tubes and kept frozen at −80°C until it was used for RNA extraction.

### RNA extraction

100 µl serum or urine was incubated at 56°C for 1 h with 0.65 mg/ml Proteinase K (Sigma P2308). Two synthetic RNAs (IDT) were spiked-in as controls before acid phenol∶chloroform extraction and then RNA was ETOH precipitated ON at −20°C. Next, DNase treatment was performed to eliminate residual DNA fragments. Finally, after a second acid phenol∶chloroform extraction, the pellet was re-suspended in DDW and two additional synthetic RNAs were spiked-in as controls.

### qRT-PCR platform [16]

RNA was subjected to a polyadenylation reaction as described previously [17]. Briefly, RNA was incubated in the presence of poly (A) polymerase (PAP; Takara-2180A), MnCl2, and ATP for 1 h at 37°C. Then, using an oligodT primer harboring a consensus sequence, reverse transcription was performed on total RNA using SuperScript II RT (Invitrogen). Next, the cDNA was amplified by real time PCR; this reaction contained a microRNA-specific forward primer, a TaqMan probe complementary to the 3′ of the specific microRNA sequence as well as to part of the polyA adaptor sequence, and a universal reverse primer complementary to the consensus 3′ sequence of the oligodT tail.

## Results

### qRT-PCR can be used to monitor low microRNA levels specifically and sensitively

**Figure 1:**
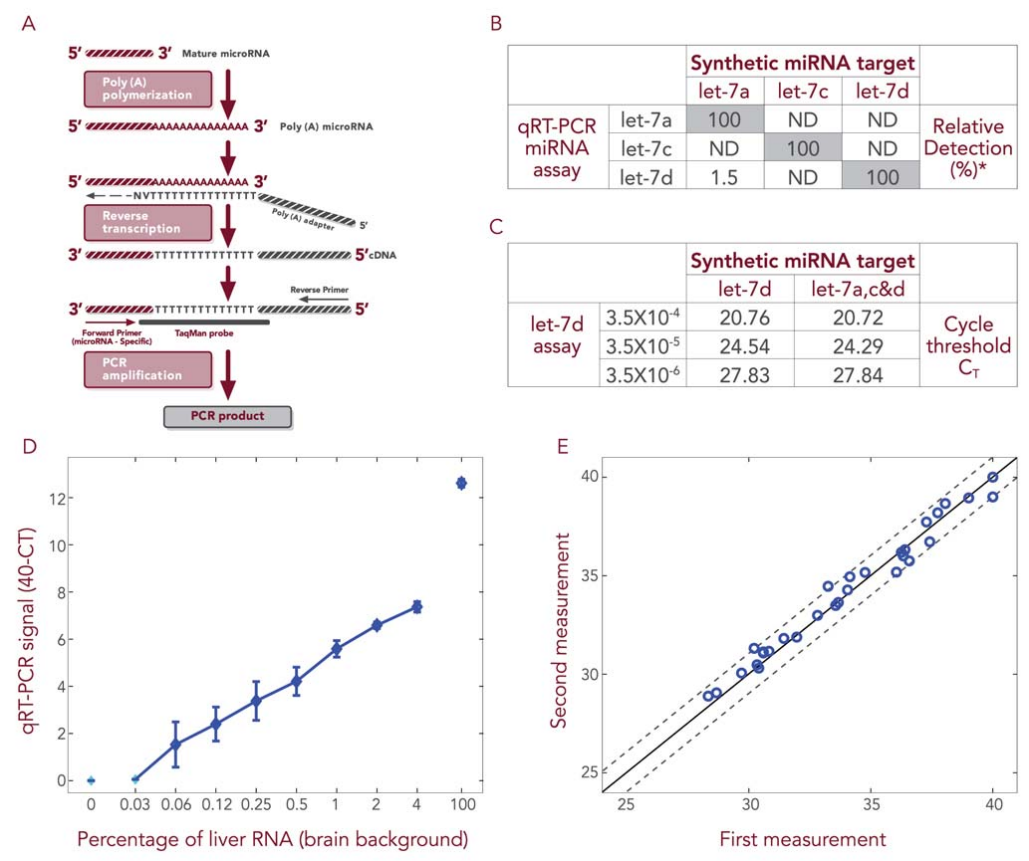
qRT-PCR can be used to monitor low microRNA levels specifically and sensitively. A) Schematic representation of the qRT-PCR method. RNA is subjected to polyA polymerase reaction. Then, a universal RT reaction is performed that allows the amplification of all microRNAs as well as mRNAs. The PCR amplification is performed using a reverse primer complementary to part of the oligodT primer and a forward primer, which is homologous to a stretch in the microRNA sequence; in addition, the amplification reaction contains a TaqMan probe that covers part of the oligodT primer sequence and some nucleotides complementary to the 3′ sequence of the microRNA. B) Each synthetic RNA (hsa-let 7a, c & d) was subjected to three independent qRT-PCR amplifications, where in each reaction there were present primers specific to only one of the three family members. PCR amplification was observed only in the reaction where the primer matches the synthetic RNA. RNA amounts are described as percentages, each relative to the level observed in the reaction containing primers matching the synthetic microRNA. ND is non-detectable. C) All three synthetic microRNAs were mixed and subjected to qRT-PCR in the presence of the let-7d primer-probe set. In parallel, the same amount of synthetic let-7d as used in the mixture was subjected alone to qRT-PCR. These parallel PCR reactions were repeated using reducing concentrations of let-7d synthetic microRNA. At all tested concentrations of let-7d, it was amplified equivalently whether alone or in the presence of homologous family members. The C_T_ of let-7d is proportional to the input microRNA amount. D) Reducing concentrations (100-0.03%) of total RNA extracted from liver tissue (Ambion Inc., No. 0360093B#) were mixed with total RNA extracted from brain tissue (Ambion Inc., No. 016P040305030A#) and the mixtures subjected to qRT-PCR, where each reaction contained a primer specific to hsa-miR-122a. Hsa-miR-122a was detected linearly, even in samples where only 0.03% liver RNA was spiked into brain RNA. When no liver RNA was introduced (100% brain RNA), hsa-miR-122a was not detected. E) The amounts of 32 different microRNAs in an RNA sample were examined on two independent occasions (by two different researchers) using the qRT-PCR platform (microRNA level is represented as C_T_ value). The two profiles were within less than 1 C_T_ difference of one another.

We have developed a proprietary qRT-PCR based platform for evaluating microRNA levels that detects specifically mature microRNA molecules (see materials and methods and Figure 1A). Initially, we validated that our platform is capable of discriminating between homologous microRNA family members that differ by only a single nucleotide. Three such microRNAs were generated synthetically (hsa-let 7a, c & d) and each one subjected to three independent qRT-PCR reactions, where in each reaction there were present PCR primers specific to only one of the 3 family members. In most of these reactions, we observed amplification only of the appropriate family member matching the specific primer, indicating that this qRT-PCR platform detects microRNA with accuracy at the single nucleotide level (Figure 1B). Next, we mixed the three synthetic microRNAs and subjected the mixture to qRT-PCR in the presence of the let-7d primer-probe set. In parallel, we took the same amount of synthetic let-7d microRNA as used in the mixture but subjected it alone to qRT-PCR. These parallel PCR reactions were repeated two further times, using reducing concentrations of let-7d synthetic microRNA. We observed that the qRT-PCR amplified let-7d equivalently whether it was alone or in the presence of homologous family members at all tested concentrations of let-7d (Figure 1C). In an additional experiment, we wished to confirm the capability of our platform to detect specific microRNAs in a biologically relevant complex background. Decreasing concentrations (from 100% down to 0%) of total RNA extracted from liver tissue were diluted into total RNA extracted from brain tissue and 0.1 ng of these mixtures subjected to PCR reaction, each reaction containing primers specific for hsa-miR-122a, a liver specific microRNA [18]. Hsa-miR-122a was detected, even when only 0.03% of liver RNA was spiked into brain RNA (Figure 1D). Importantly, hsa-miR-122a detection was linear and there was no detection in the absence of liver RNA (100% brain RNA).

Finally, to validate the reproducibility of our methods, at two independent times, two different researchers subjected the same mix of 32 microRNAs to qRT-PCR. Similar levels of abundance were determined for each microRNA on both occasions, such that the two abundance profiles (for all 32 microRNAs) were within less than 1 C_T_ difference of one another (Figure 1E). Summarily, our qRT-PCR based platform is sensitive, reproducible and specific, capable of detecting accurately a few molecules of microRNA present in a complex RNA background. Such sensitivity makes it possible to use this platform to monitor the minute amount of microRNA present in cell-free body fluids.

### microRNAs are present in cell-free bodily fluids

When we initiated this study, there had been no demonstration that microRNAs are present in cell-free body fluids. While this manuscript was in preparation, it was reported that microRNAs are detectable in serum [19]. Here, a reliable protocol is presented that we developed for extracting microRNAs from body fluids, microRNA that is cell-free and DNA-free (see Materials and Methods) and that can serve as the template in our qRT-PCR based platform.

**Figure 2:**
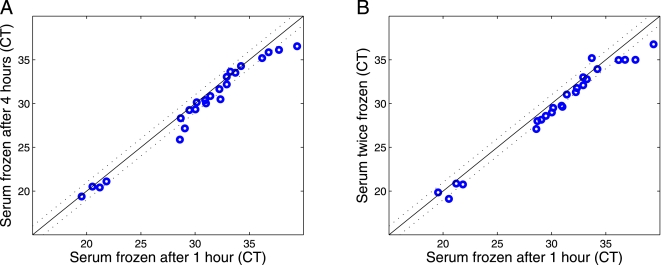
MicroRNAs are present in bodily fluids. A) microRNA levels in serum samples taken from 2 healthy individuals were measured. The levels of 18 different microRNAs (blue circles, cycle thresholds (C_T_) values) and the 4 synthetic RNA ‘spike-ins’ (in the lower left part of the graph) were found to be similar. B) To demonstrate that our extraction and evaluation methods can be applied to other body fluids, the same set of 20 microRNAs examined in serum were assessed in urine samples from 2 healthy individuals. Some microRNAs were undetectable in the urine samples and therefore are not shown. Notably, urine and serum samples demonstrate different microRNA abundance profiles.

To validate that microRNAs are indeed reproducibly detectable in serum, RNA was extracted from the sera of two healthy unrelated individuals and the levels of highly abundant microRNAs examined (Figure 2A). We observed that microRNAs are present at similar levels in both serum samples. This finding not only establishes the reproducible presence of microRNAs in serum, but also indicates that in general microRNA levels are similar among individuals. Moreover, this finding supports our premise that changes in the levels of specific microRNAs might allow detection of clinical conditions. In parallel, using the same methods we confirmed that microRNAs are also detectable in other body fluids, such as urine, saliva, amniotic fluid and pleural fluid (Figure 2B and data not shown). Of note, serum and urine display different microRNA abundance profiles as might be expected for two dissimilar biological fluids, further supporting our hypothesis that bodily fluid microRNA profiles reflect physiology.

### microRNAs in serum are sufficiently stable to serve as clinical biomarkers

**Figure 3:**
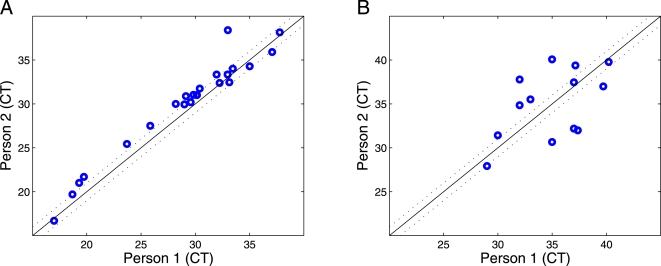
MicroRNAs are stable during serum handling. A) microRNA stability in serum samples was monitored by extracting RNA from serum samples kept for 1, 2 or 4 h at room temperature before freezing. The levels of 20 different microRNAs (blue circles), as well as of the 4 synthetic RNA ‘spike-ins’ (in the lower left part of the graph), were found to be similar across the 4 h time period. B) microRNA stability in serum samples was monitored by extracting RNA from serum samples before and after freezing. The levels of 20 different microRNAs (blue circles), as well as of the 4 synthetic RNA ‘spike-ins’ (in the lower left part of the graph), were found to be similar following re-freezing and re-thawing of the sample.

In order for microRNAs in serum to be useful as clinical biomarkers, they must be stable for reasonable periods of time, and preferably during freeze-thaw cycles, to allow for routine processing of clinical samples. Therefore, we investigated the stability of serum microRNAs at room temperature and checked the influence of freeze-thaw cycles on serum microRNA levels. We found that the levels of different microRNAs in unfrozen serum do not change substantially over a 4 hour period at room temperature (Figure 3A). Moreover, serum microRNA levels are little affected by twice freezing and re-thawing of serum samples (Figure 3B). Thus, microRNAs in serum are sufficiently robust to serve as practicable clinical biomarkers.

### Serum microRNA profiles reflect physiological conditions

**Figure 4:**
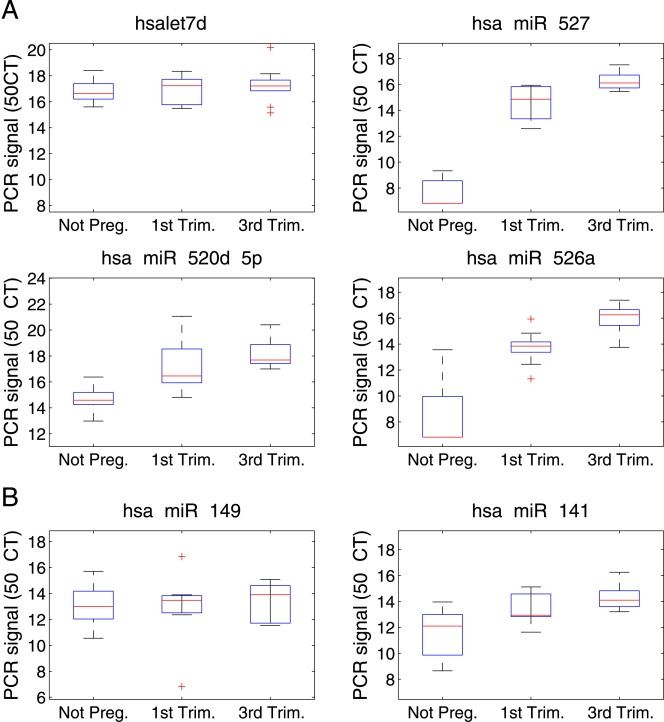
Differential amounts of four microRNAs in the sera of pregnant vs. non pregnant women. Box plots comparing microRNA levels in the sera of 10 non pregnant women (A), 10 women in the first trimester (B), and 10 women in the third trimester (C). microRNA level is specified as 50-C_T_, where C_T_ is the cycle threshold of the PCR reaction. Results were normalized by subtracting the global microRNA level in the sample (average C_T_ of the 6 microRNAs chosen for normalization) from the level (C_T_) of each microRNA. A) The three placental microRNAs (miR-527, miR-520d-5p and miR-526a) are highly abundant in the sera of pregnant women and their levels rise as pregnancy progresses. Hsa-let-7d levels are also shown; this was one of the 6 microRNAs chosen for normalization as this microRNA exhibits similar abundance across the three groups. B) microRNA miR-141 and miR-149 levels are mildly upregulated during pregnancy.

**Figure 5:**
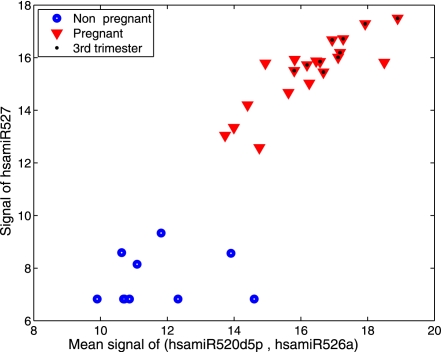
“Pregnancy classification” according to the levels of three microRNAs in the sera of pregnant vs. non pregnant women. Discrimination of pregnant women from non pregnant women based on microRNA levels in their sera. Blue circles represent non pregnant women and red triangles represent pregnant women. The location of each symbol in the plot represents the collective expression of all three microRNAs in a given serum. The y axis indicates the amount of mir-527, and the x axis indicates the average level of miR-520d-5p and miR-526a.

Finally, as a proof of concept, we investigated whether circulating microRNAs can be used to identify clinical conditions. It has been established that circulating maternal RNA contains placental embryonic RNA [2]. Therefore, we chose to compare the serum microRNA abundance profiles of non pregnant versus pregnant women, the latter in either their first or third trimester. We measured the serum levels of 28 microRNAs, including microRNAs reported to be placenta-specific [10], [20] as well as broadly expressed microRNAs. Box plots show relative microRNA levels in the sera of 10 non pregnant women, 10 women in the first trimester and 10 women in the third trimester (Figure 4A). The median fold changes in microRNA levels comparing third trimester pregnant women to non pregnant women are detailed in Table 1. MicroRNAs expressed equivalently across all samples were used for normalization. All of the placental microRNAs are found at higher levels in the sera from pregnant women, their levels rising with gestational age, and the levels of 12 microRNAs increased by more than 5-fold. Specifically, amounts of hsa-miR-526a and hsa-miR-527 are dramatically higher in the sera of third trimester pregnant women (elevated by more than 600 fold). Indeed, we found that the levels of three placental microRNAs (hsa-miR-526a, hsa-miR-527 and hsa-miR-520d-5p) could be used to accurately distinguish pregnant from non pregnant women (Figure 5).

**Table 1:** Serum microRNA levels - comparison between non pregnant women and pregnant women in their third trimester.

microRNA	delta C_T_	fold change	p-value
**hsa-miR-526a**	9.44	694	2.10E-07
**hsa-miR-527**	9.28	622	1.20E-14
**hsa-miR-515-5p**	9	511	6.90E-08
**hsa-miR-521**	7.36	164	8.10E-09
**hsa-miR-523**	4.81	28	2.20E-06
hsa-miR-524*	4.81	27	2.80E-03
**hsa-miR-518a-3p**	3.61	12	1.80E-04
**hsa-miR-520d-5p**	3.1	8.6	3.30E-07
**hsa-miR-525-3p**	2.73	6.6	5.60E-04
**hsa-miR-526c**	2.47	5.5	1.10E-01
hsa-miR-519e*	2.38	5.2	1.30E-04
**hsa-miR-518d**	2.35	5.1	7.60E-03
**hsa-miR-524**	2.27	4.8	3.80E-03
**hsa-miR-512-3p**	2.16	4.5	1.90E-03
**hsa-miR-141**	2	4.0	3.90E-04
**hsa-miR-519d**	1.9	3.7	2.60E-02
hsa-miR-517*	1.82	3.5	5.80E-02
**hsa-miR-518e**	1.5	2.8	3.50E-02
**hsa-miR-145**	0.98	2.0	3.20E-02
**hsa-miR-149**	0.92	1.9	6.00E-01
**hsa-let-7d**	0.59	1.5	5.20E-01
**hsa-miR-16**	0.39	1.3	6.90E-01
**hsa-miR-126**	0.16	1.1	1.60E-01
**hsa-miR-572**	0.11	1.1	8.70E-01
**hsa-miR-202**	0.1	1.1	4.80E-01
**hsa-miR-451**	−0.13	0.91	8.40E-01

For each microRNA, “delta C_T_” indicates the difference in median C_T_ between the serum of pregnant women in the third trimester (n = 10) and non-pregnant women (n = 10). For each sample, the relative amount of the microRNAs was normalized by subtracting the average C_T_ of the non-placenta-specific microRNAs. The fold change is the ratio of the median abundance in linear space, equal to the exponent (base 2) of the delta C_T_. P-values are calculated by a two-sided unpaired t-test.

While we were preparing these results for publication, another research group reported that hsa-miR-141 and hsa-miR-149 are present in serum and display increased abundance during pregnancy [19]. Therefore, we examined also the levels of these two microRNAs in our serum samples. We found hsa-miR-141 and hsa-miR-149 levels to be higher by 4 and less than 2 fold, respectively, in third trimester pregnant women relative to non pregnant women (Table 1 and Figure 4B). These two microRNAs are associated with epithelial tissues [16] that are not unique to pregnancy and this may explain the small changes in their amounts during pregnancy, which are insufficient to discriminate pregnant from non pregnant women.

## Discussion

The results of this study clearly uphold our basic hypothesis that microRNAs are present in bodily fluids and represent useful clinical biomarkers. Importantly, we have developed reliable methods for extracting microRNAs from bodily fluids and for evaluating their abundance. Moreover, we have demonstrated that microRNA serum levels reflect physiological conditions, such as pregnancy, and can even be used to determine pregnancy stage.

We identified previously a large subset of microRNAs that are expressed almost exclusively in the placenta [10], [20] and found that a sizeable number of these are primate-specific [10]. The biological role of these placental microRNAs remains unclear, although it is tempting to speculate that they contribute to specific morphological placental features characteristic of primates and key to their evolution [21]. Future studies may reveal how these placental microRNAs influence primate physiology. Here we show that many of these microRNAs are found in maternal serum, at levels that increase with gestational age. Easily accessible biomarkers for pregnancy complications and for various diseases are an urgent goal. In particular, preeclampsia is a disorder affecting 5–8% of pregnancies that is a leading global cause of maternal and infant illness or death [22], [23]. The recently reported, distinctive expression of microRNAs in the placenta in association with preeclampsia [24] highlights the possibility that serum levels of particular microRNAs may serve as future diagnostic biomarkers for preeclampsia.

This proof of concept study reveals the potential of body fluid microRNAs to serve as practicable molecular markers for diverse physiological and pathological conditions, especially those where microRNAs have already been found to play a critical role, such as cancer. Moreover, we demonstrate here the ease and reliability of determining body fluid microRNA profiles and thus, pave the way for their wide application, both in the research laboratory and in the clinic.
